# Role of the P2X7 receptor in *in vitro* and *in vivo* glioma tumor growth

**DOI:** 10.18632/oncotarget.27106

**Published:** 2019-08-06

**Authors:** Letícia Scussel Bergamin, Marina Capece, Erica Salaro, Alba Clara Sarti, Simonetta Falzoni, Mery Stéfani Leivas Pereira, Marco Antônio De Bastiani, Juliete Nathali Scholl, Ana Maria O. Battastini, Francesco Di Virgilio

**Affiliations:** ^1^ Graduate Program in Biological Sciences/Biochemistry, Institute of Basic Health Sciences and Department of Biochemistry, Institute of Basic Health Sciences, Federal University of Rio Grande do Sul, Porto Alegre, Brazil; ^2^ CAPES Foundation, Ministry of Education of Brazil, Brasília DF, Brazil; ^3^ Department of Morphology, Surgery and Experimental Medicine, Section of Pathology, Oncology and Experimental Biology, University of Ferrara, Ferrara, Italy

**Keywords:** cancer, glioma, P2X7R, purinergic signaling, extracellular ATP

## Abstract

Human glioblastoma cells are strikingly refractory to ATP-stimulated, P2X7 receptor (P2X7R)-mediated cytotoxicity. To elucidate the mechanistic basis of this feature, we investigated P2X7R-dependent responses in wild type and P2X7R-transfected U138 cells. Mouse GL261 glioma cells were used as an additional control. Here, we report that wild type U138 glioma cells expressed the P2X7R to very low level. Contrary to human U138 cells, mouse GL261 cells showed strong P2X7R expression and P2X7R-dependent responses. Transfection of wild type *P2RX7* into U138 cells fully restored P2X7R-dependent responses. *P2RX7* transfection conferred a negligible *in vitro* growth advantage to U138 cells, while strongly accelerated *in vivo* growth. *In silico* analysis showed that the *P2RX7* gene is seldom mutated in specimens from glioblastoma multiforme (GBM) patients. These observations suggest that the P2X7R might be an important receptor promoting GBM growth.

## INTRODUCTION

Glioblastoma multiforme is the most common malignant tumor of the central nervous system in adults, median survival being only 12 to 15 months after diagnosis [1]. GBM is characterized by rapid cell proliferation, high invasiveness, and resistance to apoptosis [1]. Histopathologically, GBM shows several necrotic foci, neovascularization, and a large inflammatory infiltrate [1, 2]. Progression of GBM is reported to be a multifactorial process consisting of numerous genetic and pathophysiological alterations that have been recently suggested to involve dysregulation of purinergic signaling [3–5].

While extracellular ATP is normally undetectable in healthy tissues, substantial amounts, up to a few hundred micromoles/liter, are found in the tumor microenvironment (TME) [6–8]. ATP is released into the TME by cancer cells or by infiltrating inflammatory cells in response to cell damage, hypoxia, mechanical stress, or stimulation by cytokines or damage-associated molecular patterns (DAMPs) [7, 9–11]. The biological effects of extracellular ATP are mediated by P2Y metabotropic (P2Y1,2,4,6,11–14) and P2X ionotrophic (P2X1–7) receptors [12, 13]. Among P2X receptors, the P2X7R subtype exhibits unique molecular, functional and pathophysiological features.

Participation in crucial processes such as inflammation and cancer makes P2X7R a focus of current hot interest. P2X7R activation by low ATP concentrations reversibly opens a cation (Na^+^, K^+^, Ca^2+^)-permeable membrane channel [7, 14, 15], that is thought to be related to cell survival [16, 17]. On the other hand, sustained stimulation or challenge with high ATP concentrations induces formation of a large conductance pore permeable to hydrophilic high molecular weight molecules (up to 900Da). Opening of this large conductance pore is thought to be mainly coupled to the activation of cell death pathways [11]. Therefore, the P2X7R is a bi-functional receptor that, depending on the level of activation, supports tumor growth or triggers cell death [6].

Contrasting data have been published on the effect of P2X7R blockade on glioma growth [18]. Recent studies have demonstrated that stimulation of P2X7R in glioma cells increases *in vitro* cell proliferation, migration and expression of MCP-1, IL-8 and VEGF, factors involved in tumor growth and metastatic spreading [4, 19, 20]. Accordingly, P2X7R pharmacological blockade inhibits C6 glioma *in vivo* growth [21]. On the other hand, it has also been reported that P2X7R pharmacological inhibition or silencing increased *in vitro* and *in vivo* C6 glioma growth [22]. Furthermore, *in vitro* proliferation of mouse GL261 glioma cells was shown to be inhibited by high concentrations of ATP or BzATP [23, 24]. Furthermore, the U138 human glioma cell line, contrary to most other cell types, many tumors included, exhibited a striking resistance to cytotoxicity mediated by extracellular ATP [25]. Given this contrasting evidence, we set to verify whether the P2X7R plays a role in U138 and GL261 glioma growth *in vitro* and *in vivo*, and to identify the factors responsible for different *in vitro* and *in vivo* P2X7R effects. Our data show that the U138 glioma cells express at low level a defective P2X7R, and that reconstitution of U138 cells with a functional wild type P2X7R affords a strong *in vivo* growth advantage.

## RESULTS

### Characterization of P2X7R in human and mouse glioma cells

**Figure 1:**
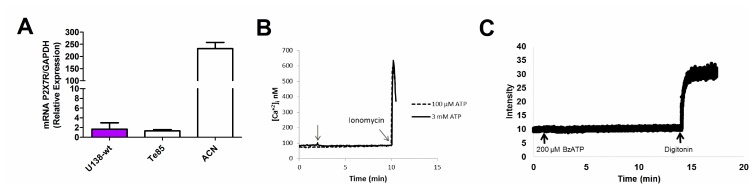
Characterization of the P2X7R in the U138-wt glioma cell line. (**A**) P2X7R mRNA expression was determined by qPCR (the ACN was used as a positive control, while the Te85 was used as negative control for P2X7R). Data are averages ± SD from triplicate samples. (**B**) Representative trace showing intracellular calcium increment following stimulation (arrow) with 100 μM or 3 mM ATP. (**C**) Representative trace showing ethidium bromide uptake after addition of 200 μM BzATP. Traces are representative of at least three similar experiments.

Human glioma tumors, notably the U138 glioma cell line, contrary to most other cell types, many tumors included, exhibit resistance to cytotoxicity mediated by extracellular ATP [25]. We thus investigated the basis of this striking phenomenon. P2X7R mRNA was barely detectable in U138 wild type (U138-wt) cells, at levels much lower than those found in other human tumor cell lines such the ACN neuroblastoma, here used as a positive control [26], and comparable to the levels found in the Te85 osteosarcoma cell line, previously characterized in our laboratory for low P2X7R expression [27] (Figure 1A). Accordingly, stimulation with a range of ATP concentrations, from 100 μM to 3 mM, did not trigger a Ca^2+^ increase, suggestive of lack of activation of the P2X7R (Figure 1B). Likewise, we were unable to detect responses suggestive of large pore opening (e.g. ethidium bromide uptake, Figure 1C).

**Figure 2:**
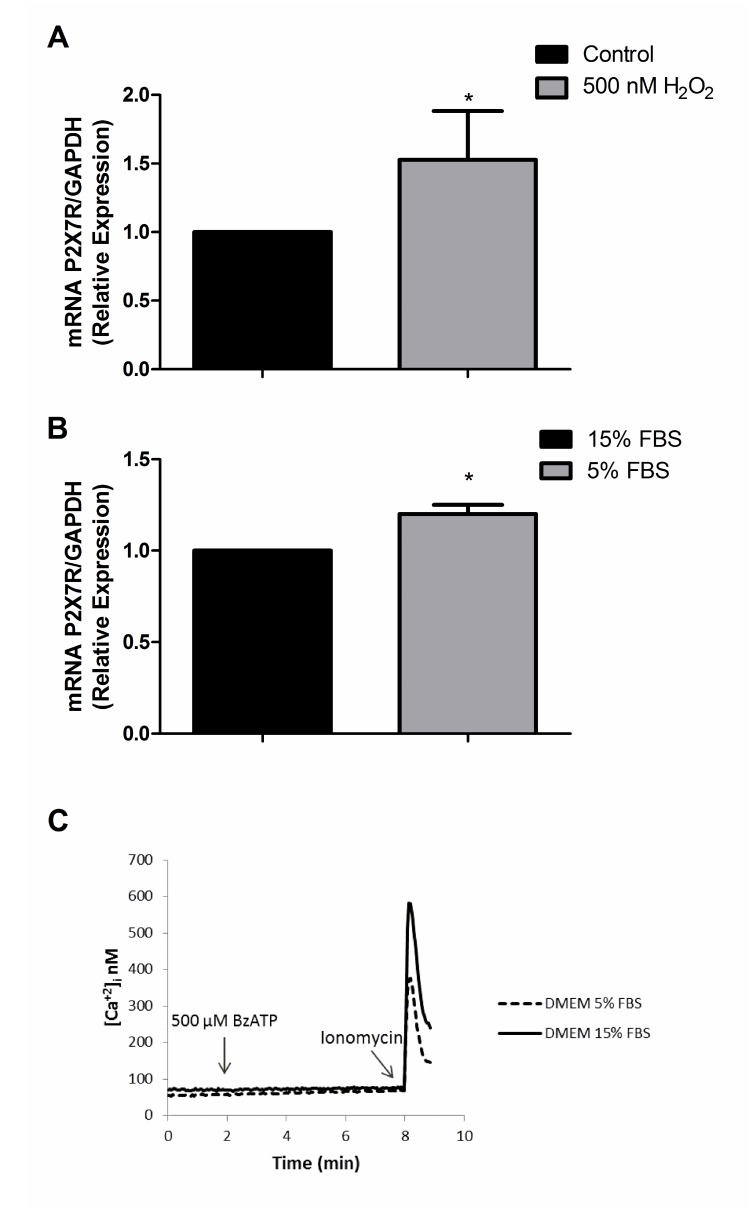
P2X7R mRNA expression after exposure to H_2_O_2_ and 5% FBS DMEM. P2X7R mRNA expression was determined by qPCR after 24 h of treatment with (**A**) 500 nM H_2_O_2_ (**B**) 5% FBS DMEM. Data are averages ± SD from at least three similar experiments. Data were analyzed by Student’s *t*-test ^*^Significantly different from control (no H_2_O_2_ or cells cultured in 15% FBS), *p* <0.05. (**C**) Representative trace showing intracellular calcium increase following stimulation with 500 μM BzATP.

Since it has been reported that P2X7R expression may change under stress conditions [28, 29], we tested whether oxidative stress (exposure to 500 nM H_2_O_2_ for 24 h) or serum deprivation (24 h culture in 5% FBS-containing serum) might restore P2X7R signaling. Oxidative stress or serum deprivation increased P2X7R mRNA levels, even by 53% in the case of oxidative stress (Figure 2A, 2B), but again no functional responses could be recorded (Figure 2C). These data suggest that U138-wt cells express very low P2X7R levels, and that even the few receptors expressed might be hypo-functional. There is precedent for the expression of non-functional P2X7R by cancer cells, whether due to loss-of-function single nucleotide polymorphisms (SNPs) [30] or to as yet poorly characterized splice variants [31]. We then verified whether U138-wt glioma cells might have *P2RX7* loss-of-function SNPs. This was indeed the case, because we found that U138-wt cells had the (1513A˃C) loss-of-function SNP. Taken together, these results show that U138-wt cells express a non-functional P2X7R and provide an explanation for refractoriness to ATP-mediated cytotoxicity, as previously reported by Battastini and coworkers [25], and explain why even after challenge with stressing agents functional responses were not recovered despite increased expression of P2X7R mRNA.

**Figure 3:**
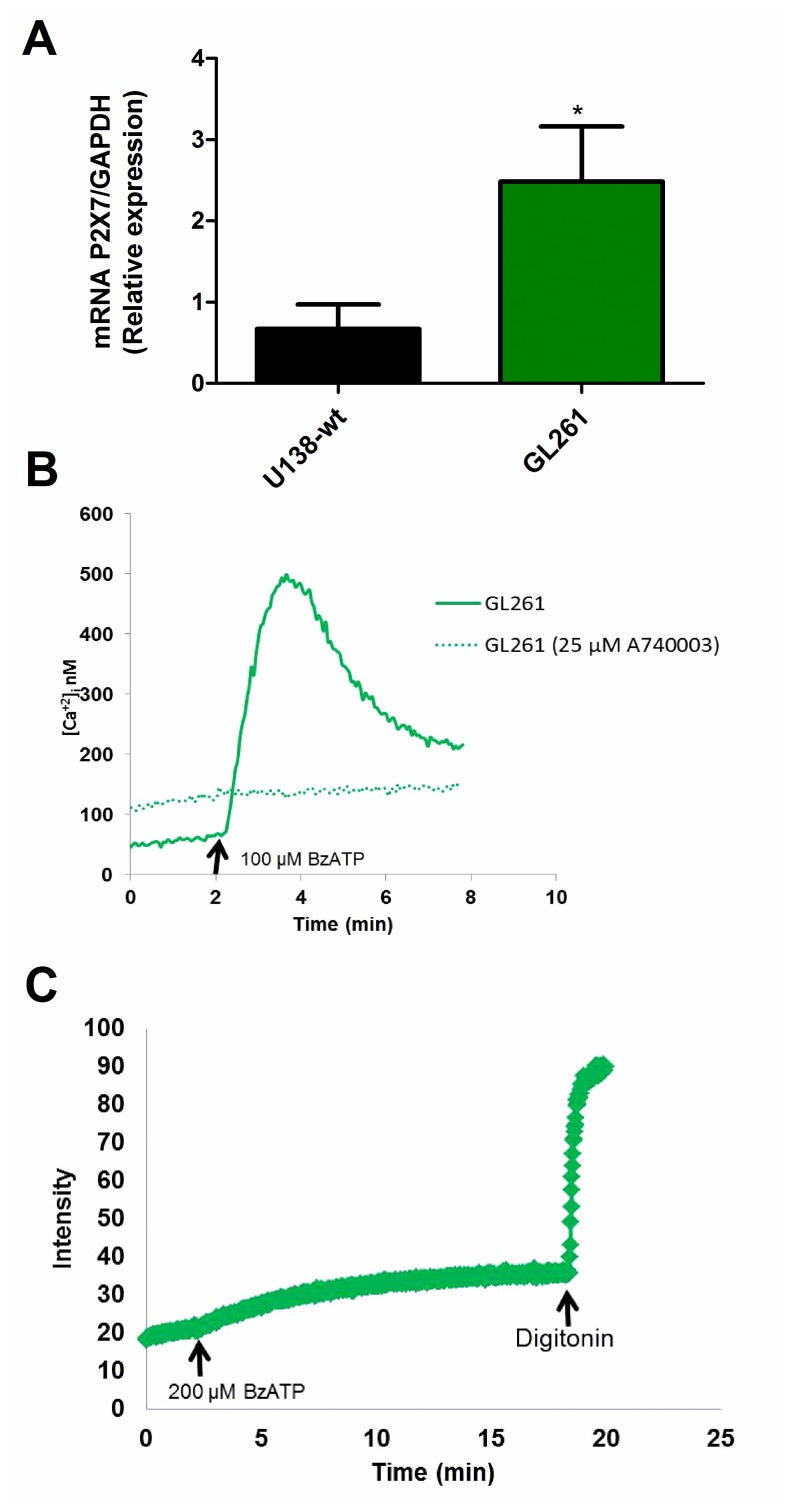
Characterization of the P2X7R receptor in mouse GL261 glioma cells. (**A**) P2X7R mRNA expression was measured by qPCR. Human U138-wt cells were used for comparison. Data are averages ± SD from at least three similar experiments analyzed by Student’s *t*-test. *p* <0.05. ^*^Significantly different from U138-wt. (**B**) Representative trace showing intracellular calcium increase following stimulation with 100 μM BzATP in presence or absence of 25 μM A740003. (**C**) Representative trace showing ethidium bromide uptake triggered by stimulation with 200 μM BzATP. Traces are representative of at least three similar obtained in different occasions.

To further understand the role that the P2X7R might play in glioma tumor biology, we investigated the mouse GL261 glioma cell line. Real time PCR (qPCR) showed that P2X7R mRNA expression was higher in GL261 versus U138-wt cells (Figure 3A). Accordingly, both Ca^2+^ mobilization and ethidium bromide uptake were stimulated by BzATP in the GL261 cell line (Figure 3B, 3C). To support the involvement of the P2X7R, Ca^2+^ uptake was fully blocked by the selective antagonist A740003 (Figure 3B).

### Transfection of *P2RX7* A/A restores P2X7R-dependent responses in human glioma cells

**Figure 4:**
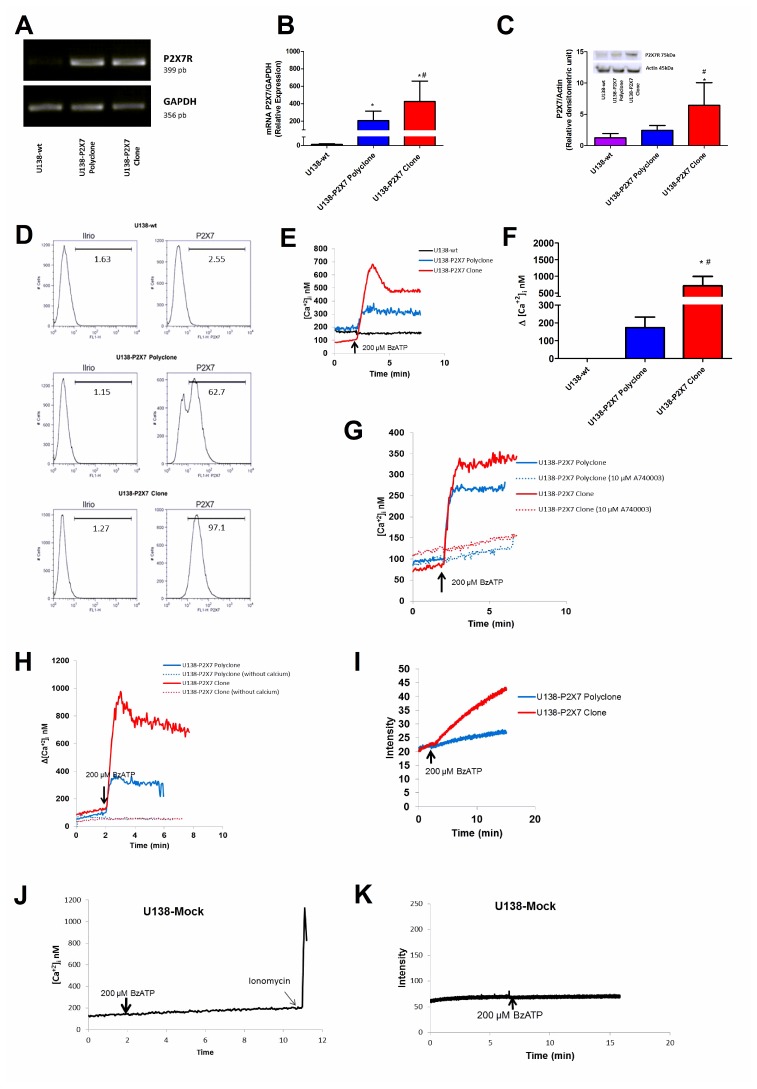
Characterization of P2X7R-mediated responses in U138-wt, U138-P2X7 polyclone and U138-P2X7 clone glioma cells. P2X7R mRNA expression was determined in transfected cells by (**A**) RT-PCR and (**B**) qPCR. P2X7R protein expression level was measured by Western blot (**C**) and flow cytometry (**D**). Representative traces (**E**) and average (**F**) intracellular Ca^2+^ rise following stimulation with 200 μM BzATP. (**G**) Representative trace showing the effect of 10 μM A740003 on the intracellular calcium rise triggered by 200 μM BzATP. (**H**) Representative trace showing the effect of extracellular Ca^2+^ removal on the BzATP-triggered intracellular Ca^2+^ rise. (**I**) Representative trace showing 200 μM BzATP-induced ethidium bromide uptake. (**J**) Representative trace showing intracellular Ca^2+^ increase and (**K**) ethidium bromide uptake following stimulation with 200 μM BzATP. Data were analyzed by ANOVA followed by Tukey test. *Significantly different from U138-wt and ^#^ significantly different from the U138-P2X7 polyclone (*p* < 0.05). All experiments were carried out at least three times, except flow cytometry (*n* = 1).

U138-wt glioma cells express very low level of P2X7R. Thus, we asked whether functional responses in this cell type might be modified by transfecting a fully functional *P2RX7* (i.e. *P2RX7* A/A). After transfection, we obtained two U138 cell populations, a U138-P2X7 polyclone, and a U138-P2X7 clone with intermediate and high P2X7R expression, respectively (Figure 4A, 4B). Western blot and flow cytometry confirmed that transfected cells had increased plasma membrane P2X7R protein levels when compared to the wild type U138 cells (Figure 4C, 4D). P2X7R-transfected cells showed a robust [Ca^2+^]_i_ increase in response to BzATP, higher in the U138-P2X7 clone than in the polyclone, which was inhibited by incubation in the presence of A740003, or by removal of extracellular Ca^2+^ (Figure 4E–4H). Both the P2X7R clone and the polyclone showed enhanced ethidium bromide uptake upon BzATP stimulation, but this response was several fold larger in the U138-P2X7 clone than in the U138-P2X7 polyclone (Figure 4I). Stimulation of mock-transfected U138 cells (U138-mock) with 200 μM BzATP did not trigger a Ca^2+^ increase or large pore opening (Figure 4J, 4K).

### Growth kinetics of P2X7R-transfected U138 glioma cells

**Figure 5:**
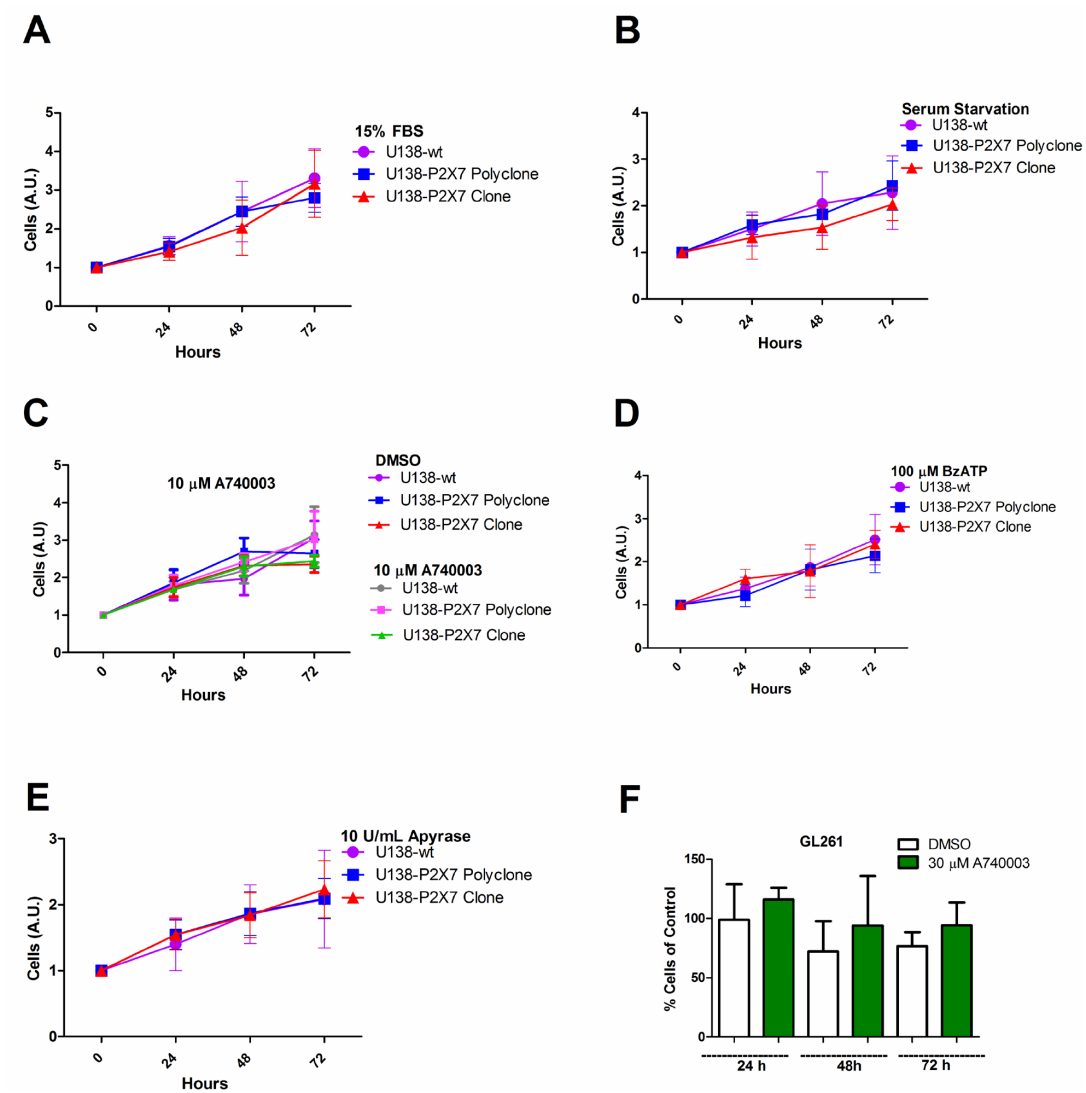
P2X7R expression does not affect *in vitro* glioma cell proliferation in low glucose DMEM. Cell proliferation was analyzed in U138-wt cells, U138-P2X7 polyclone and U138-P2X7 clone incubated for 24 h, 48 h and 72 h in DMEM containing 1 g/L glucose (low glucose) under the following conditions: (**A**) 15% FBS DMEM; (**B**) FBS-free DMEM; (**C**) absence or presence of 10 μM A740003 in FBS-free DMEM; (**D**) in the presence of 100 μM BzATP in FBS-free DMEM; (**E**) in the presence of 10 U/mL apyrase in FBS-free DMEM. In panel F the effect of A740003 (30 μM) in GL261 cultured in 15% FBS DMEM is shown. Data were analyzed by ANOVA followed by Tukey test (*p* < 0.05). All experiments were carried out at least three times.

**Figure 6:**
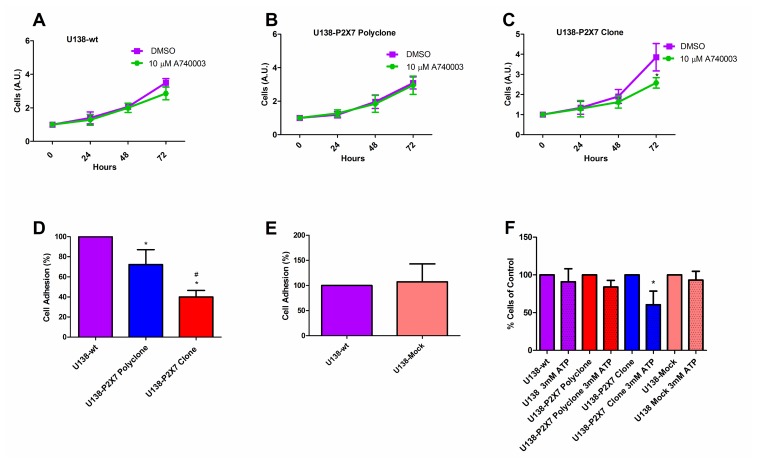
The P2X7R promotes *in vitro* glioma cell proliferation in high glucose DMEM. Cell proliferation was analyzed in (**A**) U138-wt, (**B**) U138-P2X7 polyclone and (**C**) U138-P2X7 clone cells incubated in 15% FBS-supplemented DMEM containing 4.5 g/L glucose (high glucose) for 24 h, 48 h and 72 h in the presence or absence of A740003 (10 μM). Data were analyzed by Student’s *t*-test. ^*^Significantly different from DMSO (*p* < 0.05). (**D**) Cell adhesion was evaluated as described in Materials and Methods. ^*^Significantly different from U138-wt and ^#^ U138-P2X7 polyclone (*p* < 0.05) by ANOVA followed by Tukey test. (**E**) Adhesion of U138-wt and U138-mock cells. Data were analyzed by Student’s *t*- test. (**F**) Effect of 24 h incubation in the presence of 3 mM ATP on cell viability. *Significantly different from control groups (*p* < 0.05 by ANOVA). All experiments were carried out at least three times.

The P2X7R supports cell survival and proliferation [16, 26, 32], thus we verified whether *P2RX7* transfection afforded a growth advantage to U138 cells. *In vitro* growth rates of U138-wt, U138-P2X7 polyclone and U138-P2X7 clone and GL261 were very similar, whether under normal culture conditions or under serum deprivation (Figure 5A–5F). Only when U138 cells were cultured in high glucose, a small contribution of the P2X7R was unveiled as cell proliferation was slightly decreased by addition of A740003 (Figure 6A–6C). Contrasting observations have been reported on the ability of the P2X7R to alter the expression of cell adhesion molecules [33, 34], thus we verified whether *P2RX7* transfection changed the adhesion properties of U138 cells. As shown in Figure 6D, *P2RX7* transfection reduced U138 adhesion, to a higher extent in the U138-P2X7 clone which expresses higher P2X7R levels. No difference was observed between U138-wt and U138-mock (Figure 6E). Incubation of U138 cells with 3 mM ATP significantly decreased cell proliferation in U138-P2X7 clone, thus confirming that the P2X7R was functional (Figure 6F).

### P2X7R expression increases *in vivo* glioma tumor growth

**Figure 7:**
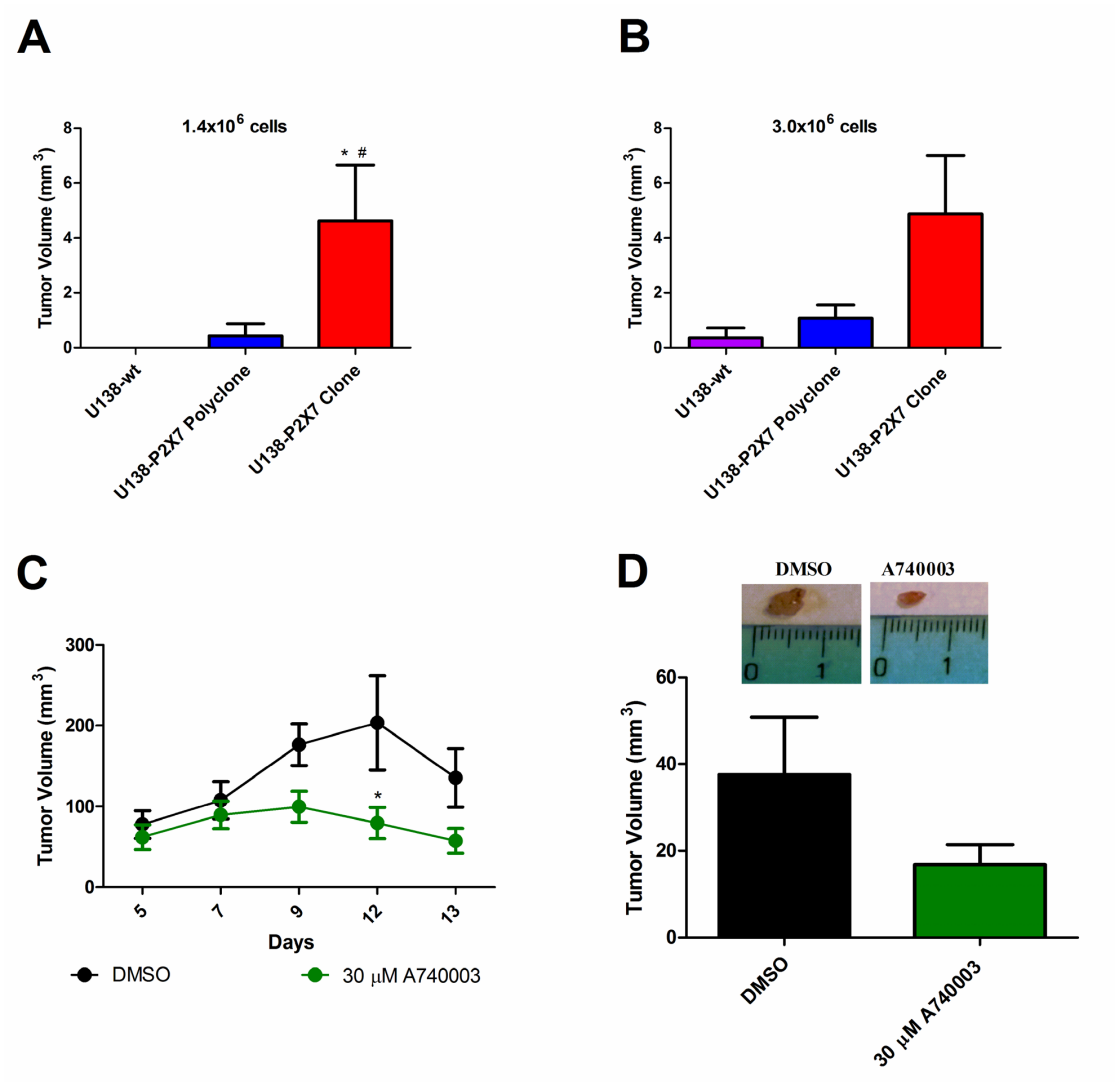
P2X7R expression increases *in vivo* tumor growth. Male athymic mice were subcutaneously inoculated with (**A**) 1.4 × 10^6^ or (**B**) 3.0x10^6^ U138-wt, U138-P2X7 polyclone or U138-P2X7 clone cells. Tumor volume was either measured in excised tumors on p.i. day 15, or *in vivo* measured by caliper. Values are mean ± S.E.M. Data were analyzed by ANOVA followed by Tukey test *Significantly different from U138-wt and ^#^ significantly different from the U138-P2X7 polyclone (*p* < 0.05) (*n* = 3–6 animals per group). GL261 cells were subcutaneously inoculated in mice and the 30 μM A740003 was intramass-administered. (**C**) Size of *in vivo* tumors. Values are shown as mean ± S.E.M. Data were analyzed by ANOVA followed by Tukey test. *Significantly different from DMSO (*n* = 4–5 animals per group). (**D**) The tumor volume of excised tumors at day 15 post-inoculum (4 animals per group). The values are presented as mean ± S.E.M. Data were analyzed by Student’s *t*- test.

The P2X7R has been shown to be a key determinant of *in vivo* growth of several human and mouse tumors [26, 32, 35]. Wild type U138, U138-P2X7 polyclone or U138-P2X7 clone glioma cells were subcutaneously inoculated into nude/nude athymic male mice in two amounts (1.4 × 10^6^ or 3.0 × 10^6^), and tumor development followed for fifteen days (Figure 7A, 7B). U138-wt cells had little tumorigenic potential as the 1.4 × 10^6^ inoculum did not produced a tumor, while with the 3.0 × 10^6^ inoculum only one mice showed a tumor of very small size. On the contrary, the U138-P2X7 polyclone produced a detectable tumor with both the 1.4 × 10^6^ and 3.0 × 10^6^ inoculum. Finally, the U138-P2X7 clone generated a tumor mass that at post-inoculum (p.i.) day fifteen was 3–4 fold larger than that generated by the U138-P2X7 polyclone. The strong acceleration of *in vivo* growth afforded by P2X7R transfection was at odd with the negligible *in vitro* growth advantage, suggesting that P2X7R expression by tumor cells molds the tumor microenvironment in such a way to promote tumor growth [35] (Figures 5 and 6).

Finally, we investigated whether *in vivo* growth of the mouse GL261 cell line was affected by P2X7R targeting. As shown by Figure 7C–7D, intramass injection of 30 μM A740003 starting at p.i. day 5 almost abrogated GL261 tumor growth, showing the key role of the P2X7R.

### *P2RX7* mutations in human GBM samples and other lines

**Figure 8:**
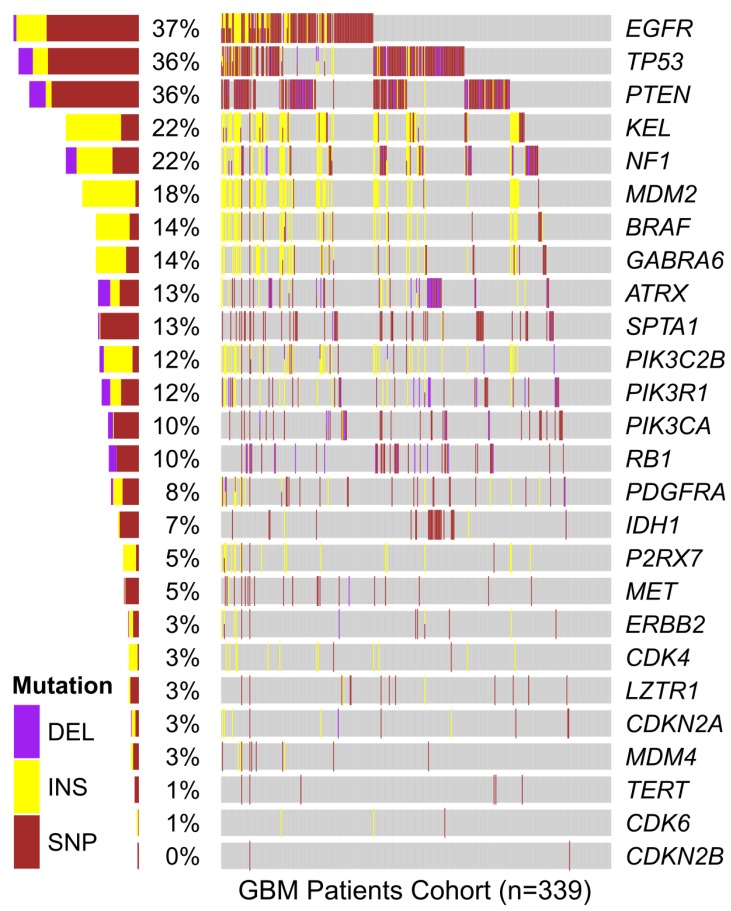
*In silico* analysis of genetic mutations found in GBM samples from the TCGA cohort. The presence of mutations in the genes listed vertically is specified by color for each of the 339 individuals in the cohort (represented horizontally along the gray bar). Total percentage of deletions (DEL), insertions (INS) and single nucleotide polymorphisms (SNP) verified in the *P2RX7* gene and other genes mutated in GBM is specified on the left side. In *P2RX7,* two types of missense mutation (g.chr12:121593912C>G and g.chr12:121603192C>T), one type of splice site (g.chr12:121613190G>T), one type of silent (g.chr12:121622170G>A) and three types of frameshift insertions (rs61745024; g.chr12:121613270_121613271insT and g.chr12:121622164_121622165insC) were identified. Data were analyzed using a free software environment for statistical computing and graphics (R Project).

**Figure 9:**
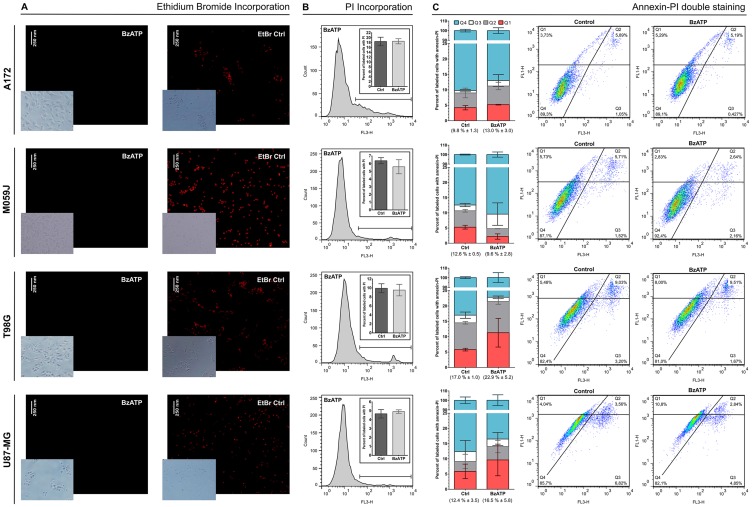
Effect of BzATP stimulation on A172, M059J, T98, and U87-MG glioma cell lines. (**A**) Ethidium bromide (EtBr) uptake after incubation for 10 min with 200 μM BzATP. (**B**) Analysis of propidium iodide (PI) incorporation after incubation for 15 min with 200 μM BzATP and a histogram representation of this group. (**C**) Analysis of annexin–PI double staining and Dot Plot representation. The values in parentheses represent the sum of Q1, Q2 and Q3 in each group (mean ± SD). Q1 = early apoptotic cells; Q2 = late apoptotic cells; Q3 = necrotic cells; Q4 = viable cells. 1 × 10^4^ events were acquired for PI and annexin-PI assays. Data were analyzed for statistical significance (*p <* 0*.*05) by Student’s *t*-test (*n* = 2–5).

In order to verify if mutations in *P2RX7* gene were related to GBM aggressiveness, a meta-analysis of genomic data from 339 GBM samples deposited in the platform “The Cancer Genoma Atlas (TCGA)” was performed. *In silico* data showed mutations in *P2RX7* gene only in 5% of biopsies, and no 1513A>C (rs3751143) SNP were detected (Figure 8). In other GBM cell lines the 1513A>C SNP was found in heterozygosity in T98G cells, while A172, M059J and U87-MG GBM cell lines showed a *P2RX7* A/A genotype. To better understand the influence of P2X7R on GBM biology, we investigated the effect of BzATP stimulation on these cell lines (Figure 9). A brief exposure to 200 μM BzATP was unable to cause ethidium bromide uptake and propidium iodide incorporation in these cells suggesting lack of large pore opening (Figure 9A and 9B). Likewise, a 24 h exposure to 200 μM BzATP failed to cause cell death by necrosis or apoptosis in these GBM lines (Figure 9C).

## DISCUSSION

The treatment of choice for GBM is surgery followed by radiotherapy and chemotherapy, but these therapies have shown limited success in increasing patient survival [1, 36]. This has prompted the search for alternative and more efficient therapies and novel tumor targets. Recently, the P2X7R has attracted great interest for its role in sustaining tumor cell autonomous growth and for its ability to modulate immune cell responses [6]. However, some studies casted doubts on the suitability of the P2X7R as a novel druggable target in glioblastoma [18]. In view of the increasing emphasis placed on the role of purinergic signaling in tumor progression, in the present work we investigated the effect of P2X7R expression on *in vitro* and *in vivo* glioma cell growth.

Both P2Y and P2X receptors can modulate the intracellular Ca^2+^ concentration in different ways. Activation of P2X receptors promotes Ca^2+^ entry from the extracellular space [37], while stimulation of P2Y receptors activates phospholipase C, inositol-phosphate formation, and the release of Ca^2+^ from intracellular stores [38]. U138-wt cells were remarkably refractory to ATP or BzATP stimulation, and accordingly expressed negligible levels of the P2X7R, that were increased by exposure to cell-stressing conditions such as H_2_O_2_ or nutrient deprivation. Availability of wt and P2X7R-transfected U138 cell clones enabled us to closely verify the role played by this receptor in glioma cell growth. The P2X7R might in principle oppose tumor cell proliferation, by virtue of its potent cytotoxic effect when overstimulated, or support tumor progression due to its growth-promoting activity [6, 39]. Transfection of P2X7R affected some essential phenotypic features of U138 cells, such as adhesion, but had no substantial effect on *in vitro* proliferation, confirming previous data showing no effect on U251 human glioma cells or human glioblastoma stem cells exposed to selective P2X7R antagonists or to apyrase [19, 40].

It is well known that *in vitro* experiments represent a very rough approximation to the multi-factorial process of *in vivo* tumor growth [41], and in addition this study was entirely performed in GBM cell lines, except for in *silico* data shown in Figure 8. Prolonged exposure of many tumor cells to high concentrations of P2X7R agonists usually promotes cell death *in vitro*, but this is unlikely to reflect *in vivo* conditions occurring in the TME [7, 32]. The TME is very heterogeneous, consisting of cancer cells, endothelial cells, fibroblasts and immune cells. Within this complex milieu, P2X7R-mediated responses not only affect tumor growth directly (cell autonomous effects), but also indirectly by modulating the activity of anti-tumor or immunosuppressive immune cells [42–44], or by stimulating release of growth factors (e.g. VEGF) [32]. Thus, interpretation of our *in vivo* experimental models needs to be taken with important caveats. Ectopic inoculation of the tumor in the mouse flank, as opposed to the orthotropic implant, does not reproduce the appropriate pathophysiological conditions of brain tumors. In addition, human U138 glioma cell growth was investigated *in vivo* in immunocompromised mice, a model far from the pathophysiologically relevant condition, although controls were also run with GL261 mouse glioma cells in the syngeneic host. P2X7R-transfected U138 glioma cells showed *in vivo* higher proliferation rate, and more importantly became fully sensitive to the growth-inhibiting activity of P2X7R-blockers, like the mouse GL261 glioma cells which express a fully functional P2X7R. The advantage conferred by *P2RX7* transfection was striking because wild type U138 cells were barely able to implant, and only at the higher inoculum (3.0x10^6^ cells), and the polyclone proliferated at a rate several fold lower than the U138-P2X7 clone. In line with this hypothesis, Morrone and collaborators [2006] have demonstrated that ATP, presumably acting at the P2X7R, plays an important role for cell implantation and initial tumor growth, since the co-injection of apyrase with C6 glioma cells in an *in vivo* glioma model promoted a reduction in the glioma size [41].

The human *P2RX7* is highly polymorphic, numerous single nucleotide polymorphisms having been identified. One of these, the 1513A˃C SNP, which causes the substitution of glutamate for alanine at position 496 in the intracellular C-terminal tail, is responsible for reduced activity and defective transfer to the plasma membrane of the P2X7R [45]. Besides expressing very low P2X7R levels, U138-wt cells also bore a defective 1513A˃C SNP, that further hindered ATP-mediated responses and explained why, despite stressing agents increased P2X7R expression, they did not restore P2X7R-dependent responses. The T98G lineage presented a *P2RX7* A/C genotype, while the others (M059J, A172 and U87-MG) showed wild type *P2RX7* A/A. Although wild type 1513 A/A supports a functional P2X7R, these cell lines, like U138-wt cells are refractory to BzATP stimulation. As a clear demonstration of the specificity of the ATP or BzATP response, full responses to these nucleotides were restored by transfection of U138 cells with a human wild type P2X7R. The mouse GL261 glioma, on the other hand, expressed a fully functional P2X7R, as previously shown [23, 24]. However, even in human glioma cells the P2X7R might be uncoupled from cell death, as Tamajusuku *et al*. [2010] showed that in U87-MG cells the P2X7R was expressed at the protein level but was not sufficient to trigger ATP-dependent cell death [23]. Another study showed in this same GBM line that treatment with BzATP far from causing cell death, promoted proliferation and migration via stimulation of the MEK/ERK pathway [20]. In conclusion, these results show that expression of the P2X7R in GBM is not by itself sufficient to make these cell susceptible to ATP/BzATP-mediated cytotoxicity, whether because they might express the defective C allele, or because of low level of P2X7R expression. Taken together, these results support previous studies by Battastini and coworkers [5, 19, 23, 25] showing glioma cells refractoriness to ATP-mediated cytotoxicity.

In order to verify if the presence of mutations in the *P2RX7* gene can be used as prognostic markers in GBM, a meta-analysis of gene expression data from TCGA-GBM biopsies was performed. Only 5% of the biopsies showed mutations in this gene, and no sample showed the SNP 1513A˃C SNP. However, RNA seq data from the same source (TCGA) showed that P2X7R is strongly up-regulated in both glioma and GBM [34]. Non-synonymous SNP in the P2X7R have been variably associated to several disease conditions hematopoietic tumors included [46]. However, while there consensus on the finding that most malignant tumors overexpress the P2X7R [6], the possible role of gain- or loss-of-function SNPs is less clear. The *P2RX7* gene has been recently identified as a susceptibility gene for glioma in dogs [47], but the picture is as yet unclear in humans [34, 47]. Loss-of-function SNPs in the P2X7R might promote growth by protecting cancer cells from apoptosis [48], but on the other hand gain-of-function SNPs might enhance tumor cell proliferation and support release of trophic factors such as VEGF, or of immunosuppressive mediators such TGF-β [6]. Together, these results suggest that *P2RX7* mutations are rare in GBM tumors, and likely of negligible diagnostic or prognostic significance, especially when compared to the frequency of mutations in other genes [49–51]. On the contrary, overexpression of P2X7R might provide useful diagnostic and therapeutic hints.

## MATERIALS AND METHODS

### Cell cultures and transfections

The U138 human glioma cell line was obtained from Cell Lines Service (Eppelheim, Germany), mouse GL261 glioma cell line was a generous gift from Doctor Steve Lacroix (Department of Anatomy and Physiology, Université Laval, Quebec, Canada) and A172, T98G, M059J and U87-MG human glioma cell lines were obtained from American Type Culture Collection (ATCC). U138 and GL261 lines were maintained in Dulbecco’s modified Eagle’s medium (low or high glucose) (DMEM; Sigma-Aldrich, St. Louis, MO, USA), as indicated in the different experiments described below, supplemented with 15% (v/v) fetal bovine serum (FBS; EuroClone, Milan, Italy) and penicillin (EuroClone), and streptomycin (EuroClone). A172, T98G, M059J and U87-MG lines maintained under the same conditions, however using 10% (v/v) FBS. Human U138 glioma cells at 80% confluence were transfected with Mirus (Mirus Bio LLC, Madison, WI, USA), according to the manufacturer’s instructions. We used pcDNA3.1 containing the human *P2RX7* gene sequence. Stable human U138-mock, U138-P2X7 polyclone and U138-P2X7 clone were kept in the continuous presence of 0.4 mg/mL G418 sulfate (Geneticin; Calbiochem, La Jolla, CA, USA).

### P2X7R polymorphism analysis

Genomic DNA was extracted from U138-wt with a Genomic DNA tissue kit, according to the manufacturer’s instructions (Macherey-Nagel, Duren, Germany). In the A172, T98G, M059J and U87-MG, genomic DNA was extracted using the Illustra Blood GenomicPrep Mini Spin Kit (GE Healthcare Life Sciences, Marlborough, MA, USA).

The 1513A˃C (E496A) SNP was analyzed in genomic DNA samples with the TaqMan MGB probe technique, as previously described [52]. Allele specific hybridization was detected using a StepOne™ Real-Time PCR system (Applied Biosystems, Foster City, CA, USA). The genotype was designated by allelic discrimination through analysis of the fluorescent signal during and at the end of DNA amplification.

### RT-PCR and qPCR

For the GL261, U138-wt, U138-P2X7 polyclone and U138-P2X7 clone, RNA was isolated using TRIzol Reagent (Thermo Fisher Scientific, Waltham, CA, USA) and the PureLink RNA Mini Kit (Thermo Fisher Scientific, Waltham, CA, USA). RNA was added to each cDNA synthesis reaction using the High Capacity cDNA Reverse Transcription Kit (Applied Biosystems).

RT-PCR was performed in a total volume of 20 μL, which included cDNA, each forward and reverse primer (Foward hP2X7R: 5′-AGATCGTGGAGAATGGAGTG-3′; Reverse hP2X7R: 5′-TCCTCGTGGTGTAGTTGTGG-3′; Forward hGAPDH: 5′-CGACCACTTTGTCAAGCTCA; Reverse hGAPDH: 5′-AGGGGTCTACATGGCAACTG-3′), and PCR master mix (Thermo Fisher Scientific). The reference gene glyceraldehyde 3-phosphate dehydrogenase (GAPDH) was used as an endogenous control.

Real Time PCRs were carried out in the AB StepOne Real Time PCR (Applied Biosystems) with TaqMan Gene Expression Master Mix (Applied Biosystems) using the following primers: for U138 cells, P2X7R (Hs00175721-m1; Applied Biosystems) and GAPDH (4326317E; Applied Biosystems); for GL261 cells, P2X7R (Mm00440578; Applied Biosystems), mouse GAPD (GAPDH) endogenous control (VIC^®^/MGB probe, primer limited; Applied Biosystems). All results were analyzed by the 2^-Δ/ΔCT^ method [53].

### Western blot

Total U138-wt, U138-P2X7 polyclone and U138-P2X7 clone cell lysates were prepared in lysis buffer (300 μM sucrose, 1.0 mM K_2_HPO_4_, 5.5 mM glucose, 20 mM HEPES) supplemented with protease inhibitors (1 mM phenylmethylsulfonyl fluoride, 1 mM benzamidine, all from Sigma-Aldrich). Proteins were quantified using the Coomassie blue method [54]. Appropriate amounts of protein were resolved on Bolt Mini Gels 4–12% gels (Life Technologies). After electrophoresis, proteins were transferred to nitrocellulose membranes (GE Healthcare Life Sciences, Milan, Italy). Membranes were incubated overnight with the primary antibody (Ab) at 4°C. The anti-P2X7R antibody (cat. no. P 9122; Sigma-Aldrich) was used at a diluition of 1:500 in 1% BSA (Sigma-Aldrich). The anti-actin antibody (cat. no. MA5-11869, Thermo Fisher Scientific) was diluted 1:5000 in 5% non-fat milk. Nitrocellulose membranes were incubated with the corresponding horseradish peroxidase-conjugated secondary antibody at a 1:3000 dilution.

### Cytosolic free calcium concentration measurements

The cytosolic Ca^2+^ concentration was measured using the fluorescent Ca^2+^ indicator Fura-2-acetoxymethyl ester (Fura-2/AM) (Thermo Fisher Scientific) [55]. Cells were incubated at 37° C for 20 minutes in sodium solution (125 mM NaCl, 5 mM KCl, 1 mM MgSO_4_, 1 mM NaH_2_PO_4_, 20 mM HEPES, 5.5 mM glucose, 1 mM CaCl_2_, pH 7.4) supplemented with 4.0 μM Fura-2/AM and 250 μM sulfinpyrazone (Sigma-Aldrich). In experiments performed in the absence of external Ca^2+^, CaCl_2_ was omitted and 1.0 mM EGTA was added._._ Cells were kept at 37° C in a thermostat-controlled and magnetically-stirred cuvette of a Cary Eclipse Fluorescence Spectophotometer (Agilent Technologies, Milan, Italy) and [Ca^2+^]_i_ was determined at the 340/380nm excitation ratio and at 505 nm emission wavelengths. ATP (Sigma-Aldrich), BzATP (Sigma-Aldrich) or A740003 (Tocris Bioscience, Bristol, UK) were added when indicated.

### Ethidium bromide uptake

Changes in plasma membrane permeability on exposure to BzATP (200 μM) (Sigma-Aldrich) were studied by ethidium bromide uptake. One hundred thousand cells were kept at 37° C in a thermostat-controlled and magnetically stirred cuvette of a Cary Eclipse Fluorescence Spectophotometer (Agilent Technologies) in the presence of 20 μM ethidium bromide (Sigma-Aldrich). Fluorescence changes were acquired at 360 nm and 580 nm excitation and emission wavelengths, respectively.

For A172, M059J, T98G, U87MG, 8 × 10^4^ cells were seeded in 96-well plates for 24 h, washed with PBS, incubated for 10 min in PBS supplemented with ethidium bromide (5 μg/mL) and 200 μM BzATP. A positive control of ethidium bromide incorporation was performed treating cultures with permeable buffer solution during ethidium bromide exposure (3.5 mM trisodium citrate, 0.5 mM Tris buffer and 0.1 % nonidet). Ethidium bromide fluorescence was observed using a inverted fluorescence microscope (Nikon Eclipse TE300; Melville, NY, USA). The exposure time used to acquire images from treated samples was the same used for acquisition of images from permeabilized cells (maximal ethidium bromide uptake).

### Flow cytometry

For flow cytometry analysis, 2 × 10^6^ U138-wt, U138-P2X7 polyclone and U138-P2X7 clone cells were washed twice with PBS and centrifuged. Pellets were suspended and incubated with the anti-P2X7R L4 monoclonal antibody (kindly provided by Professor James Wiley, Florey Neuroscience Institutes, University of Melbourne, Australia) for 30 min at 4° C. Next, all samples were washed with PBS and incubated for 30 min with secondary FITC-conjugated rabbit anti-mouse antibody (Dako, Milan, Italy). Labeled cells were then washed with PBS and immediately measured in a FACSCalibur Flow Cytometer (BD Biosciences, Franklin Lakes, NJ, USA) and data were analyzed using FlowJo Software (TreeStar, Ashland, CA, USA).

The other human GBM cell lines were seeded in 12-well plates (A172 and M059J cells at a density of 4 × 10^4^ cells/well, T98G and U87-MG cells at a density of 6 × 10^4^ cells per well), and treated on the second day for 24 h with 200 μM BzATP. After this time, annexin V-FITC–propidium iodide double staining was verified using Annexin V-FITC Apoptosis Detection Kit I (BD Biosciences) according to the manufacturer’s instructions. Labeled cells were measured in FACSCalibur Flow Cytometer (BD Biosciences) and data were analyzed using FlowJo Software. For propidium iodide (PI) incorporation in glioma cells, 1 × 10^5^ cells were incubated with PI during 15 min of exposure to 200 μM BzATP, and then washed with PBS. PI-positive cells were counted by flow cytometry.

### Proliferation assay

U138-wt, U138-P2X7 polyclone and U138-P2X7 clone were seeded and, once at the subconfluence stage, were treated with 100 μM BzATP, a P2X7R agonist, (Sigma-Aldrich), or 10 U/mL apyrase, an ATP and ADP scavenger (Sigma-Aldrich), or 10 μM A740003, a selective P2X7R antagonist (Tocris Bioscience), or DMSO (0.01%) (Sigma-Aldrich) in low glucose (1000 mg/L D-glucose), FBS-free, DMEM. Proliferation rate was also measured in low glucose, 15% FBS-supplemented, DMEM. Pictures were taken at different time points (0 h, 24 h, 48 h and 72 h) with a phase-contrast Olympus microscope (Olympus Life Science Europe, Hamburg, Germany) and then analyzed with the ImageJ software [26]. In other experiments, cells were maintained in high glucose (4500 mg/L D-glucose), 15% FBS-supplemented DMEM, and treated with 10 μM A740003 or DMSO (0.1%). At the 0 h, 24 h, 48 h and 72 h time points, cells were stained with crystal violet and cell proliferation was analyzed by optical density (OD, 595 nm) in a microplate reader. In parallel, to check the effect of P2X7R stimulation on cell growth, U138-wt, U138-mock, U138-P2X7 polyclone and U138-P2X7 clone were incubated in low glucose, 15% FBS-supplemented DMEM in the presence of 3 mM ATP (Sigma-Aldrich). Twenty four h after, cells were rinsed, stained with crystal violet and cell proliferation analyzed by optical density.

GL261 cells were maintained in low glucose, 15% FBS-supplemented DMEM, seeded in 24-well plate and allowed to grow to semi-confluence. Cells were then treated with 30 μM A740003, for 24 h, 48 h and 72 h. After treatment, cells were trypsinized, and counted in a hemocytometer by the Trypan blue exclusion test.

### Adhesion assay

U138-wt, U138-mock, U138-P2X7 polyclone and U138-P2X7 clone were seeded in 96-well plates and incubated for 2 h at 37° C. Non-adherent cells were removed by carefully washing three times with PBS. Adherent cells were fixed with 4% paraformaldehyde (PFA) and stained with crystal violet. Cell adhesion was analyzed by measuring optical density (OD) in a microplate reader [56].

### *In vivo* experiments

One million four hundred thousand or 3.0 × 10^6^ U138-wt, U138-P2X7 polyclone or U138-P2X7 clone cells were diluted in 100 μL of PBS and then inoculated into the dorsal flank of 5- to 7-week-old nude/nude male mice (Harlan Laboratories, Correzzana, Italy). Animals were examined at p.i. day 5, 7, 9, 12, 13, 14 and 15 to assess general health conditions and evaluate tumor growth. All animals were euthanized by cervical dislocation on day 15 and tumors were excised (*n* = 3–6 animals per group).

Seven hundred and fifty thousand GL261cells were diluted in 100 μL of PBS and then injected into the dorsal flank of male, C57Black/6 mice. Animals were examined at p.i. day 5, 7, 9, 12, 13, 14 and 15, and were treated with 30 μM A740003 or DMSO directly administered in the tumor mass. At day 15, all animals were euthanized by cervical dislocation and the tumors were excised (*n* = 4–5 animals per group).

Tumor size was measured and the volume calculated according to the following equation: volume = π/6 [w1 × (w2)^2^], where w1= greater tumor mass diameter and w2= smaller tumor mass diameter. Animal procedures were approved by the University of Ferrara Ethic Committee and the Italian Ministry of Health in compliance with international laws and policies (European Economic Community Council Directive 86/109, OJL 358, Dec. 1, 1987, and NIH Guide for the Care and Use of Laboratory Animals).

### *In silico* experiments

Glioblastoma biopsies were collected and processed according to the guidelines established by The Cancer Genomic Atlas (TCGA), a platform that provides RNA sequencing and gene mutations data from various types of cancer for meta-analyzes (https://cancergenome.nih.gov/) [57]. The R statistical environment [58] was used to analyze gene expression data (RNAseq) of 339 human tumor biopsies. Table 1 shows the clinical characteristics of GBM cohort used for meta-analyzes. For a comparative study, genes that according to the literature are usually mutated in biopsies of glioblastomas were also analyzed in this cohort [49–51].

**Table 1:** Clinical characteristics of TCGA GBM cohort

*n* (M/F)	Race			Age				Survival (years)				
	Caucasian	Other	NI	Min.	Max.	Mean	Median	Min.	Max.	Mean	Median	% alive in 3 years
339 (209/130)	309	27	3	21	89	59,85	60 (52–69)	0,01	10,63	1,15	0,87 (0,39–1,49)	3,4

Abbreviations: NI, not-identified.

### Statistical analysis

Data were analyzed for statistical significance by Student’s *t*-test or one way ANOVA followed by a post-hoc test for multiple comparisons (Tukey test). The data are expressed as the mean±S.D. (*in vitro* experiments) or mean±S.E.M. (*in vivo* experiments). Differences were considered significant at *p<*0 *.*05.
